# A Dual-Source Evidence–Driven Semi-Supervised Belief Rule Base for Fault Diagnosis

**DOI:** 10.3390/s26082444

**Published:** 2026-04-16

**Authors:** Xin Zhang, Zhiying Fan, Wei He, Huafeng He

**Affiliations:** 1 High-Tech Institute of Xi’an, Xi’an 710025, China; zhangxin_shanxi@163.com (X.Z.); hhf0903@163.com (H.H.); 2 School of Computer Science and Information Engineering, Harbin Normal University, Harbin 150025, China; 2024300703@stu.hrbnu.edu.cn

**Keywords:** belief rule base (BRB), fault diagnosis, semi-supervised learning, pseudo-labeling

## Abstract

In the fault diagnosis of complex industrial systems, labeled samples are expensive to obtain, which leads to insufficient training data for the belief rule base (BRB) model. Although unlabeled samples are abundant, the uncertainty of their pseudo-labels may undermine semi-supervised learning and hinder accurate parameter optimization of the BRB model. To address these issues, a dual-source evidence-driven semi-supervised BRB method (SS-BRB) is proposed for fault diagnosis. The proposed method makes effective use of unlabeled samples while preserving the interpretability and inference transparency of the BRB model. To improve the reliability of pseudo-labels in semi-supervised learning, a dual-source evidence-driven pseudo-labeling mechanism is designed. In this mechanism, local similarity information is combined with the global inference results of the BRB model. An entropy factor and a feature distance factor are introduced to adaptively adjust the confidence of pseudo-labels. In this way, the quality of pseudo-labels is improved, and the influence of noisy samples is reduced. Based on this mechanism, high-confidence pseudo-labeled samples are incorporated into the training set to further optimize the model. Experimental results show that the proposed method achieves good diagnostic performance on both the gearbox dataset and the WD615 diesel engine dataset. Even with limited labeled data, the proposed method still achieves high accuracy, robustness, and good generalization performance.

## 1. Introduction

Complex systems usually have large-scale structures, closely coupled functions, and wide fault coverage. Timely and accurate fault diagnosis can reduce the losses and improve system reliability and safety. It can also optimize maintenance strategies and extend system service life. Therefore, research on fault diagnosis is of great significance [1,2].

At present, fault diagnosis methods can be mainly divided into four categories [3,4].

Physics-based methods establish mathematical models by analyzing the internal mechanisms and operating laws of a system. Key features are then extracted and clearly represented. For example, Huang et al. [5] used an improved hidden Markov model to classify faults in motor drive systems. Jafari et al. [6] proposed an inter-turn fault detection method based on modal current and indicators. These methods can achieve high diagnostic accuracy when the fault mechanism is well characterized. However, they rely heavily on accurate mathematical modeling and expert knowledge, which limits their application in complex industrial systems with uncertain operating conditions. More importantly, such methods are generally developed for fully labeled scenarios and cannot effectively utilize unlabeled samples in semi-supervised diagnosis. They also do not provide explicit mechanisms to identify or suppress noisy pseudo-labels. When the actual fault patterns cannot be sufficiently represented by the predefined physical model, their diagnostic performance may degrade.

Data-driven methods train models using large amounts of data and enable self-learning and fault prediction. For instance, Chen et al. [7] combined multiscale attention entropy with random forests for fault diagnosis of rotating machinery. Chao et al. [8] integrated physical models with deep learning to improve diagnostic accuracy under varying operating conditions. These methods can automatically learn discriminative features and often achieve strong predictive performance. However, most data-driven models require sufficient labeled samples for stable training. Under limited labeled data conditions, semi-supervised extensions usually depend on pseudo-labeling strategies, in which incorrect pseudo-labels may be gradually reinforced during iterative model updating. In addition, most of these models are black boxes and lack interpretability. As a result, it is difficult to determine whether an unlabeled sample is sufficiently supported by the current model, which weakens model robustness when pseudo-label noise exists or when the sample distribution is sparsely covered.

Knowledge-based methods perform reasoning and diagnosis by using expert experience or prior knowledge. For example, Netzer et al. [9] improved diagnostic efficiency by formalizing domain knowledge. Chen et al. [10] adopted a modular fault tree method to reduce the complexity of analysis. These methods have the advantage of clear reasoning logic and good interpretability. However, they are constrained by expert knowledge and predefined rules, making them difficult to adapt to dynamic system changes. In semi-supervised scenarios, they usually do not provide a data-dependent mechanism to evaluate the reliability of pseudo-labels. When unlabeled samples fall outside the effective scope of existing knowledge rules, these methods cannot provide sufficient evidence for reliable diagnosis or training guidance.

Semi-quantitative information methods combine qualitative and quantitative analysis to address complex system problems. For example, Ozyurt et al. [11] developed a semi-quantitative fault monitoring and diagnosis framework by generating episodic fuzzy rules from numerical behavior envelopes. Eltahan et al. [12] introduced a fuzzy-logic-based semi-quantitative risk assessment method for crude oil shipping pump failures in a petroleum production unit. These studies demonstrate that semi-quantitative information methods can integrate heterogeneous information and handle uncertainty effectively in industrial systems. Among them, the belief rule base (BRB), as a representative semi-quantitative information method, is especially suitable for fault diagnosis because it can combine expert knowledge with data and provide interpretable reasoning results [13].

Fault features in industrial complex systems are highly uncertain. Therefore, a diagnostic model must be able to handle uncertain information and integrate heterogeneous data [14]. However, the above physics-based, data-driven, and knowledge-based methods all have their own limitations in fault diagnosis of complex systems, especially under limited labeled data conditions. In comparison, semi-quantitative information methods, particularly BRB, show clear advantages in uncertainty handling, heterogeneous information fusion, and inference interpretability. Nevertheless, existing BRB methods still face significant challenges under semi-supervised learning conditions [15,16].

On the one hand, the quality of pseudo-labels for unlabeled samples is difficult to guarantee. In industrial fault diagnosis, the data often suffer from noise, class overlap, and blurred boundaries. Under limited labeled data conditions, pseudo-labels generated for unlabeled samples may be inaccurate. Once such noisy pseudo-labels are directly incorporated into BRB training, they may distort belief distributions, damage rule consistency, and reduce the effectiveness of parameter optimization. On the other hand, some unlabeled samples may fall into regions with insufficient rule coverage or weak rule activation. In such cases, the current BRB model cannot provide sufficiently strong inference support, and the resulting training signals may be unreliable, which further affects the generalization ability of the model. Existing BRB methods usually lack an explicit mechanism to jointly evaluate pseudo-label reliability from multiple evidence sources and to identify whether a sample is sufficiently supported by the current rule base.

To address these problems, a semi-supervised BRB learning framework is proposed for fault diagnosis tasks under limited labeled data conditions. The framework improves diagnostic performance by effectively exploiting unlabeled data while preserving rule interpretability and inference transparency. To reduce the impact of pseudo-label noise and alleviate the effect of insufficient rule coverage, a dual-source evidence-driven mechanism for pseudo-label reliability evaluation is designed. This mechanism combines local similarity information among samples with the global inference results of the BRB model to assess the reliability of pseudo-labels for unlabeled samples. Local similarity reflects whether an unlabeled sample is consistent with its neighborhood structure, which helps identify unreliable pseudo-labels caused by noise, class overlap, or blurred decision boundaries. Meanwhile, BRB-based global inference reflects whether the sample is sufficiently supported by the current rule base and corresponding belief distribution. By jointly considering these two sources of evidence, the proposed method can not only improve pseudo-label quality and reduce the influence of noise but also avoid directly using weakly supported samples in insufficient-coverage regions as strong supervision during training. In this way, the proposed SS-BRB overcomes the main limitations of existing BRB methods in semi-supervised fault diagnosis and achieves a better balance between unlabeled data utilization, rule consistency, and model generalization.

The main contributions of this study are summarized as follows.

(1) A semi-supervised BRB learning framework is proposed for fault diagnosis under limited labeled data conditions. The framework enables effective utilization of unlabeled samples while maintaining the transparency and interpretability of the BRB model.
(2) A dual-source evidence-driven pseudo-label evaluation and selection mechanism is developed. The proposed mechanism jointly incorporates local sample similarity and BRB-based global inference evidence to improve pseudo-label reliability, mitigate the negative impact of noisy pseudo-labels during training, and alleviate the adverse effect of insufficient rule coverage caused by weakly supported unlabeled samples.
(3) Extensive experiments conducted on the gearbox and WD615 diesel engine datasets demonstrate that the proposed method outperforms several representative comparison methods in terms of diagnostic accuracy, robustness, and generalization, thereby validating its effectiveness in semi-supervised fault diagnosis.

The rest of this paper is organized as follows. Section 2 presents the main issues in fault diagnosis addressed in this study. Section 3 introduces the dual-source evidence-driven pseudo-labeling mechanism. Section 4 describes the inference and training processes of the SS-BRB model. Section 5 verifies the effectiveness of the proposed method through two case studies. Section 6 concludes the paper.

## 2. Problem Formulation

In this section, the problems existing in industrial faults are elaborated in Section 2.1, and the SS-BRB model is constructed in Section 2.2.

### 2.1. Problem Formulation of Fault Diagnosis

The fault diagnosis of complex industrial systems faces two main problems:

**Problem** **1.** *How to train the BRB model with unlabeled samples under limited labeled data conditions. BRB model training generally depends on labeled samples. When labeled data are scarce, the BRB model cannot be sufficiently trained. Although a large number of unlabeled samples are available, they cannot be directly used for model training, which restricts further improvement in model performance. In industrial fault diagnosis, obtaining labeled data is usually costly, and therefore only a limited number of labeled samples can be collected in practice. At the same time, unlabeled samples contain abundant potentially useful information. However, without ground-truth labels, they cannot be directly incorporated into BRB parameter learning in the same way as labeled samples.*

*The model parameters of BRB are denoted as Θ. When only labeled samples are used, the model training process can be represented as:*

(1) $$\Theta^{*}=\arg\min_{\Theta }L\left(\Theta ,D_{L}\right)$$

where $\Theta^{*}$ represents the optimal parameters, and $L(\Theta ,D_{L})$ denotes the loss function based on the labeled sample set $D_{L}$. Since the unlabeled sample set $D_{U}$ does not contain true labels, the information from $D_{U}$ cannot be directly utilized in the aforementioned training process. Therefore, under the condition of limited $N_{L}$, the BRB model is prone to insufficient training.

**Problem** **2.** *How to ensure the reliability of pseudo-labels for unlabeled samples under limited labeled data conditions. Since unlabeled samples do not have ground-truth labels, pseudo-labels must be generated before they can be incorporated into training. However, pseudo-labels may be inaccurate, which can adversely affect model performance [17,18]. In industrial fault diagnosis, fault data are often affected by noise, class overlap, and ambiguous class boundaries. When labeled samples are limited, it becomes more difficult to accurately determine the categories of unlabeled samples, thereby increasing the risk of erroneous pseudo-label assignment.*

*The labeled sample set is denoted as $D_{L}={\left\{\left(x_{i},y_{i}\right)\right\}}_{i=1}^{N_{L}}$, and the unlabeled sample set is denoted as $D_{U}={\left\{x_{j}\right\}}_{j=1}^{N_{U}}$. For an unlabeled sample $x_{j}$, its pseudo-label can be expressed as:*

(2) $${\hat{y}}_{j}=\arg\max_{c}p\left(c|x_{j}\right)$$

*where $x_{i}$ represents the sample features, $y_{i}$ denotes the ground-truth label, ${\hat{y}}_{j}$ is the pseudo-label, and $p(c|x_{j})$ indicates the confidence that sample $x_{j}$ belongs to class c. $N_{L}$ and $N_{U}$ represent the numbers of labeled and unlabeled samples, respectively.*

### 2.2. Construction of the SS-BRB Model

**Figure 1:**
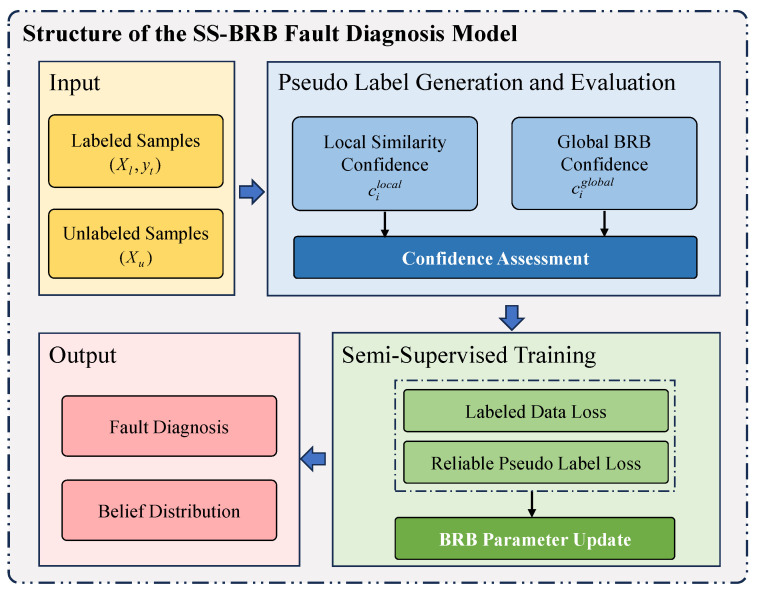
Structure of the SS-BRB fault diagnosis model.

SS-BRB is an IF-THEN rule-based system that integrates expert knowledge. It consists of a set of belief rules, where the $k_{th}$ rule can be described as follows [19]:

(3) $$\begin{array}{cc} \; & IF\;x_{1}\;is\;A_{1}^{k}\wedge x_{2}\;is\;A_{2}^{k}\wedge \ldots \wedge x_{T_{k}}\;is\;A_{T_{k}}^{k}\\ \; & THEN\;y\;is\;\left\{\left(R_{1},\beta_{1,k}\right),\ldots ,\left(R_{N},\beta_{N,k}\right)\right\}\;\left(\sum_{n=1}^{N}\beta_{n,k}\leq 1\right)\\ \; & \mathrm{With}\;\mathrm{rule}\;\mathrm{weight}\;\theta_{k},\;k\in \left\{1,2,\ldots ,L\right\}\\ \; & \mathrm{and}\;\mathrm{attribute}\;\mathrm{weight}\;\delta_{1},\delta_{2},\ldots ,\delta_{i},\;i\in \left\{1,2,\ldots ,T_{i}\right\} \end{array}$$

where $x_{1},x_{2},\ldots ,x_{T_{k}}$ denote the antecedent attributes, namely the diagnostic features derived from sensor measurements; $A_{1}^{k},A_{2}^{k},\ldots ,A_{T_{k}}^{k}$ are the corresponding reference values of these antecedent attributes in the $k_{th}$ belief rule; $T_{k}$ represents the number of antecedent attributes contained in the $k_{th}$ rule; $R_{1},R_{2},\ldots ,R_{N}$ denote the consequent propositions, corresponding to different fault states or operating conditions of the sensor system; $\beta_{1,k},\beta_{2,k},\ldots ,\beta_{N,k}$ are the belief degrees associated with the consequent propositions; $\theta_{k}$ is the weight of the $k_{th}$ rule; $\delta_{1},\delta_{2},\ldots ,\delta_{i}$ denote the weights of the antecedent attributes; and *L* is the total number of rules in the proposed SS-BRB model. The SS-BRB fault diagnosis model proposed in this paper is designed to address the challenge of scarce labeled samples in complex industrial systems. The structure of the model is shown in Figure 1 and is composed of the following parts:

(1) Input layer: It is composed of a labeled sample set $X_{l}=\left\{\left(x_{i},y_{i}\right)\right\}$ and an unlabeled sample set $X_{u}=\left\{x_{j}\right\}$.
(2) Pseudo-label generation and evaluation module: Pseudo-labels are generated for unlabeled samples, and their reliability is assessed through a dual-source evidence mechanism.
(3) Semi-supervised training module: Labeled samples and unlabeled samples with highly reliable pseudo-labels are utilized to train the parameters of the BRB model, including rule weights, attribute weights, and consequent belief degrees.
(4) Output layer: Fault diagnosis inference is carried out based on the trained BRB model, and a belief distribution across different fault types is provided.

The overall framework forms a closed-loop system, enabling the effective utilization of unlabeled data while preserving the interpretability and transparency of the BRB model in reasoning.

It should also be noted that the proposed SS-BRB is a reconfigurable diagnosis framework rather than a fixed rule model for a specific device. When the diagnostic target changes, the core semi-supervised learning mechanism remains unchanged, while the task-specific BRB structure is reconstructed according to the new equipment. In practice, this reconstruction includes: (1) selecting informative monitoring attributes from the candidate sensor variables using data analysis and engineering knowledge; (2) defining initial semantic reference values according to the physical meaning and statistical distribution of the selected attributes; (3) initializing the BRB rule base with a small number of labeled samples; and (4) further optimizing rule weights, attribute weights, consequent belief degrees, and rule support through the proposed dual-source evidence-driven semi-supervised learning process. Therefore, expert knowledge in this framework mainly provides an interpretable initialization prior, whereas the final model is adaptively refined through labeled and unlabeled data.

## 3. Dual-Source Evidence-Driven Pseudo-Labeling Mechanism

To improve the training stability and pseudo-label reliability of semi-supervised BRB, a dual-source evidence-driven pseudo-labeling mechanism (DSE-PL) is proposed in this paper. Highly reliable pseudo-label weights are calculated for each unlabeled sample by integrating local similarity evidence and global inference evidence from BRB.

When only local similarity is used, the evaluation of pseudo-labels relies entirely on the proximity of samples in the feature space. In high-dimensional spaces or under sparse sample distributions, the confidence calculation may be inaccurate, leading to noisy pseudo-labels and negatively affecting the semi-supervised training performance. Conversely, relying solely on BRB global inference can utilize the rule base for overall consistency judgment. However, when rule coverage is insufficient or samples lie near rule boundaries, the inference signal is limited, and the training signal may be inadequate, restricting the model’s generalization capability.

Therefore, by fusing local similarity and global inference into dual-source evidence, both local feature consistency and global rule consistency are taken into account. This not only improves the reliability of pseudo-labels but also enhances the training stability and diagnostic performance of the model under limited labeled data conditions.

In the dual-source mechanism, local similarity is calculated based on the proximity of samples in the feature space. To mitigate the “curse of dimensionality” in high-dimensional spaces and the issue of feature scale differences, a radial basis function (RBF) kernel is introduced, and feature standardization is performed [20]:

(4) $$x_{i,j}^{norm}=\frac{x_{i,j}-\mu_{j}}{\sigma_{j}},$$

(5) $$c_{i}^{local}=\frac{1}{K}\sum_{k=1}^{K}\exp\left(-\gamma {∥x_{i}^{norm}-x_{k}^{norm}∥}^{2}\right)$$

where $x_{i,j}^{norm}$ is the standardized feature vector of the sample. $\mu_{j}$ and $\sigma_{j}$ are the mean and standard deviation of the $j_{th}$ feature, respectively. *K* is the number of nearest neighbors. γ is the RBF kernel parameter. $c_{i}^{local}$ is the confidence of local similarity.

The initial BRB model is used to perform global inference on unlabeled samples. Then, the global confidence is obtained as follows:

(6) $$c_{i}^{global}=f_{\theta }\left(x_{i}\right)$$

where $f_{\theta }\left(x_{i}\right)$ is the confidence output by the BRB model. θ denotes the model parameters of the BRB model. These parameters include rule weights, attribute weights, and consequent belief degrees.

A dual-evidence fusion strategy is adopted to combine the local confidence and the global confidence. To dynamically balance the contributions of local similarity evidence and BRB-based global inference evidence, sample-wise adaptive fusion coefficients are introduced.

First, the reliability of local evidence and global evidence is quantified as:

(7) $$r_{i}^{local}=\frac{c_{i}^{local}}{d_{i}+\varepsilon },$$

(8) $$r_{i}^{global}=c_{i}^{global}\left(1-{\tilde{E}}_{i}\right)$$

where $d_{i}$ is the feature-distance factor, ${\tilde{E}}_{i}$ is the normalized entropy, and ε is a small positive constant to avoid division by zero.

Then, the adaptive fusion coefficients are defined as:

(9) $$\alpha_{i}=\frac{r_{i}^{local}}{r_{i}^{local}+r_{i}^{global}+\varepsilon },\;\beta_{i}=\frac{r_{i}^{global}}{r_{i}^{local}+r_{i}^{global}+\varepsilon }$$

with $\alpha_{i}+\beta_{i}=1$.

Accordingly, the fused confidence is calculated as

(10) $$c_{i}^{fused}=\alpha_{i}c_{i}^{local}+\beta_{i}c_{i}^{global}.$$

On this basis, an entropy factor and a feature-distance factor are further introduced to refine the final pseudo-label weights.

The entropy factor is used to measure the uniformity of the confidence distribution [21]:

(11) $$E_{i}=-\sum_{k=1}^{C}{\hat{p}}_{i,k}\log\left({\hat{p}}_{i,k}+\epsilon \right)$$

where ${\hat{p}}_{i,k}$ is the normalized confidence of sample *i* for class *k*. *C* is the number of classes. ϵ is a small constant and is introduced to avoid the logarithm of zero.

The feature-distance factor is used to measure the feature similarity between a sample and its neighbors:

(12) $$d_{i}=\frac{1}{K}\sum_{k=1}^{K}∥x_{i}^{norm}-x_{k}^{norm}∥.$$

Finally, the entropy factor and the feature-distance factor are combined to calculate the final weight of each pseudo-label. Since a larger entropy value indicates a more uniform confidence distribution and thus higher uncertainty, the normalized entropy is first defined as:

(13) $${\tilde{E}}_{i}=\frac{E_{i}}{\log C},$$

and its complementary term $\left(1-{\tilde{E}}_{i}\right)$ is used to assign larger weights to more reliable pseudo-labels. Accordingly, the final weight of each pseudo-label is calculated as:

(14) $$w_{i}^{\mathrm{final}}=\frac{c_{i}^{\mathrm{fused}}\cdot \left(1-{\tilde{E}}_{i}\right)\cdot f\left(d_{i}\right)}{\sum_{j=1}^{N}c_{j}^{\mathrm{fused}}\cdot \left(1-{\tilde{E}}_{j}\right)\cdot f\left(d_{j}\right)},$$

(15) $$f\left(d_{i}\right)=\frac{1}{1+d_{i}}$$

where $d_{i}$ denotes the average feature-space distance between sample *i* and its *K* nearest neighbors. This monotonically decreasing function assigns a larger weight to samples located in compact neighborhoods and a smaller weight to isolated samples. Therefore, pseudo-labels supported by nearby neighbors contribute more to the final confidence, while those associated with large feature-space distances are suppressed. The denominator ensures that all pseudo-label weights are normalized, satisfying $\sum_{i=1}^{N}w_{i}^{final}=1$.

The final pseudo-label confidence is calculated as:

(16) $$c_{i}^{\mathrm{final}}=w_{i}^{\mathrm{final}}c_{i}^{\mathrm{fused}}$$

where $c_{i}^{\mathrm{fused}}$ is the fused confidence of sample *i*, and $w_{i}^{\mathrm{final}}$ is the normalized pseudo-label weight determined by the entropy factor and the feature-distance factor.

The proposed dual-source evidence-driven mechanism combines local similarity with BRB-based global inference to evaluate the reliability of pseudo-labels for unlabeled samples. This enables the complementary use of local and global information. In addition, an entropy factor and a feature-distance factor are introduced to adaptively adjust pseudo-label weights. This design helps reduce the influence of noisy pseudo-labels during training. By ensuring that only highly reliable pseudo-labels are incorporated into training, the mechanism also preserves the rule interpretability and inference transparency of the BRB model.

## 4. The Fault Diagnosis Model Based on SS-BRB

In this section, the inference process of SS-BRB is described in Section 4.1, the training process of SS-BRB is presented in Section 4.2, and Section 4.3 provides the computational cost analysis.

### 4.1. Inference Process of the SS-BRB

The SS-BRB employs the evidential reasoning (ER) approach, which provides a clear and easy-to-understand framework for the integration of multi-source information [22]. With this approach, expert knowledge can be incorporated, the inference process can be made interpretable, and the influence of different pieces of evidence on the final result can be quantified [23]. The inference procedure of SS-BRB can be summarized in the following steps:

Step 1: The input observation data are transformed into a belief distribution:

(17) $$A_{i,j}\leq x_{i}\leq A_{i,j+1},\left\{\begin{array}{c} \alpha_{i,j}=\left(A_{i,j+1}-x_{i}\right)/\left(A_{i,j+1}-A_{i,j}\right)\\ \alpha_{i,j+1}=1-\alpha_{i,j}\\ \alpha_{i,j^{\prime }}=0,\;j^{\prime }=1,2,\ldots ,J_{i}\;\left(j+1\leq J_{i}\right)\;\&\;j^{\prime }\neq j,j+1 \end{array}\right.$$

(18) $$S\left(x_{i}\right)=\left\{\left(A_{i,j}^{k},\alpha_{i,j}^{k}\right)|i=1,\ldots ,T_{k},j=1,\ldots ,J_{i}\right\}$$

where $\alpha_{i,j}^{k}$ denotes the matching degree of the $j_{th}$ reference value of the $i_{th}$. $J_{i}$ denotes the number of reference values. $A_{i,j}^{k}$ and $A_{i,j+1}^{k}$ denote two adjacent reference values. $S(x_{i})$ denotes the information transformation process for the input data $x_{i}$.

Step 2: The activation weight of each rule is calculated as:

(19) $$\omega_{k}=\frac{\theta_{k}\prod_{i=1}^{T_{k}}{\left(\alpha_{i,j}^{k}\right)}^{{\bar{\delta }}_{i}}}{\sum_{l=1}^{L}\theta_{l}\prod_{i=1}^{T_{k}}{\left(\alpha_{i,j}^{l}\right)}^{{\bar{\delta }}_{i}}},\;{\bar{\delta }}_{i}=\frac{\delta_{i}}{\max_{i=1,2,\ldots ,T_{k}}\left\{\delta_{i}\right\}}$$

where $\omega_{k}$ denotes the activation weight of the $k_{th}$ rule. $\delta_{i}$ denotes the relative weight of the attribute. ${\bar{\delta }}_{i}$ denotes the normalized weight of the $i_{th}$ attribute.

Step 3: The activated rules are aggregated by the evidential reasoning algorithm:

(20) $$\beta_{n}=\frac{\mu \left[\prod_{k=1}^{L}\left(\omega_{k}\beta_{n,k}+1-\omega_{k}\sum_{j=1}^{N}\beta_{j,k}\right)-\prod_{k=1}^{L}\left(1-\omega_{k}\sum_{j=1}^{N}\beta_{j,k}\right)\right]}{1-\mu \left[\prod_{k=1}^{L}\left(1-\omega_{k}\right)\right]},$$

(21) $$\mu ={\left[\sum_{j=1}^{N}\prod_{k=1}^{L}\left(\omega_{k}\beta_{j,k}+1-\omega_{k}\sum_{j=1}^{N}\beta_{j,k}\right)-\left(N-1\right)\prod_{k=1}^{L}\left(1-\omega_{k}\sum_{j=1}^{N}\beta_{j,k}\right)\right]}^{-1}$$

where $\beta_{n}\left(n=1,2,\ldots ,N\right)$ denotes the belief degree of the $n_{th}$ outcome level $R_{n}$.

Step 4: The belief degree of the evaluation result is calculated. The aggregated result in Step 3 is mapped to the output belief degree corresponding to the input variable:

(22) $$S\left(x\right)=\left\{\left(R_{n},\beta_{n}\right)|n=1,2,\ldots ,N\right\}$$

where *x* denotes the input variable.

Step 5: The fault diagnosis result of the model is obtained:

(23) $$u\left(S\left(x\right)\right)=\sum_{n=1}^{N}u\left(R_{n}\right)\beta_{n}$$

where $u(R_{n})$ denotes the utility of $R_{n}$.

### 4.2. Training Process of the SS-BRB

**Figure 2:**
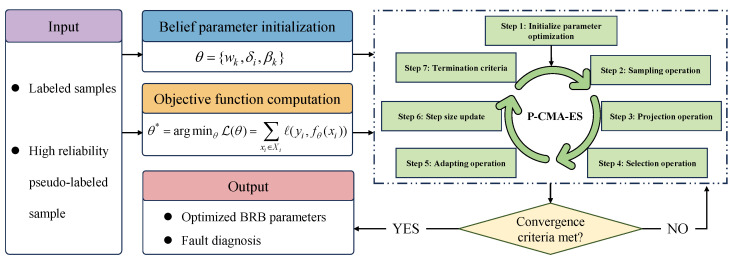
Training process of the SS-BRB model.

The training objective of the SS-BRB model is to optimize the parameter set of the BRB, including rule weights, attribute weights, and consequent belief degrees, so that high diagnostic accuracy can be achieved under the condition of limited labeled samples. During training, both labeled samples and highly reliable pseudo-labeled samples generated by the dual-source evidence mechanism are used. The detailed training flow is shown in Figure 2.

Step 1: Parameter initialization is performed. All BRB parameters, $\theta =\{w_{k},\delta_{i},\beta_{k}\}$, are initialized.

Step 2: The training objective function is defined. The objective is to minimize the joint loss of labeled samples and highly reliable pseudo-labeled samples:

(24) $$\theta^{*}=\arg\min_{\theta }L\left(\theta \right)=\sum_{x_{i}\in X_{l}}\ell \left(y_{i},f_{\theta }\left(x_{i}\right)\right)+\lambda \sum_{x_{j}\in X_{u}^{H}}\ell \left({\hat{y}}_{j},f_{\theta }\left(x_{j}\right)\right)$$

where ℓ(·) denotes the loss function, which is set as the mean squared error. λ is used to balance the contributions of labeled samples and pseudo-labeled samples. $f_{\theta }\left(\cdot \right)$ denotes the BRB inference function.

Step 3: The model parameters are optimized using the projection covariance matrix adaptation evolution strategy (P-CMA-ES). To address the nonconvex optimization problem and reduce the risk of convergence to local optima, P-CMA-ES is employed for parameter optimization [24]. In each iteration, candidate solutions are generated and evaluated according to the objective function. The mean vector and covariance matrix are then updated, and parameter projection under constraints is performed.

Step 4: The optimization proceeds iteratively until convergence. P-CMA-ES continues parameter sampling, evaluation, and updating until a stopping criterion is satisfied, such as reaching the maximum number of iterations or having the change in the loss function fall below a predefined threshold. The optimized BRB parameter set $\theta^{*}$ is thereby obtained.

Step 5: The optimized SS-BRB model is then deployed for inference. Using the optimized parameters, the model performs fault diagnosis for new samples. Benefiting from the learned rules and the training guidance provided by highly reliable pseudo-labels, the model can deliver accurate and interpretable diagnostic results.

It should be noted that the current optimization process based on P-CMA-ES is mainly designed for offline parameter training rather than strict real-time parameter updating. In practical industrial applications, the proposed framework is more suitable for an offline-training and online-inference deployment mode. Specifically, the trained SS-BRB model can be directly used for real-time fault diagnosis of newly arriving samples, while newly collected data can be accumulated in a sliding window or incremental sample buffer for subsequent model updating. In this way, online diagnosis and parameter optimization can be decoupled. Since the BRB inference process mainly involves input matching, rule activation, and evidential reasoning aggregation, its computational burden is much lower than that of the P-CMA-ES-based training process, making real-time inference more feasible in practice. For scenarios requiring continuous model adaptation without operational downtime, a more realistic extension is to perform background re-optimization periodically or in an event-triggered manner based on newly accumulated labeled or high-confidence pseudo-labeled samples, while the current deployed model remains responsible for online diagnosis. Therefore, the present work mainly validates the effectiveness of the proposed framework for semi-supervised learning and online inference after offline training, whereas continuous online parameter updating remains an important direction for future research.

### 4.3. Computational Cost Analysis

To further clarify the practical applicability of the proposed method, the computational costs of the training and inference phases are distinguished here.

In the training phase, the main computational burden comes from pseudo-label generation and P-CMA-ES-based parameter optimization. Let *N* denote the number of samples, *d* the feature dimension, ξ the population size, *p* the parameter dimension, and *G* the number of optimization iterations. The local-similarity calculation with the improved KNN introduces the main sample-level cost, while the optimization stage requires storing candidate solutions and the covariance matrix, with space complexity

(25) $$\begin{array}{c} O(\xi p+p^{2}). \end{array}$$

Therefore, the training stage is computationally more expensive and is mainly carried out offline. In contrast, after model training is completed, the inference phase only involves BRB reasoning for new samples, including input matching, rule activation, and evidential reasoning aggregation. If only $k\ll L^{\prime }$ rules are effectively activated, the inference cost is approximately proportional to the number of activated rules:

(26) $$\begin{array}{c} O(k), \end{array}$$

This indicates that the inference stage is much lighter than the training stage. Therefore, the computational characteristics of SS-BRB are consistent with the deployment mode discussed above: the current framework is suitable for offline training and online inference, while strict real-time online model updating is beyond the scope of the present study.

## 5. Case Study

To validate the effectiveness of the proposed SS-BRB method for fault diagnosis in real industrial systems, experimental studies are conducted on two typical types of equipment, namely a gearbox and a diesel engine. Through case analysis, the roles of the dual-source evidence mechanism in pseudo-label generation, semi-supervised training, and BRB inference are examined. In addition, the diagnostic accuracy and robustness of the model are evaluated under the condition of limited labeled samples. Specifically, Section 5.1 presents the experimental settings, Section 5.2 analyzes the fault diagnosis performance of SS-BRB, Section 5.3 verifies the effectiveness of the DSE-PL mechanism, and Section 5.4 summarizes the experiments in this part.

### 5.1. Experimental Setup

The gearbox is a core transmission component in complex mechanical systems. Its operating condition directly affects the performance and reliability of the whole system. Timely fault diagnosis can be used to monitor the gearbox condition in real time, detect potential faults in advance, and enable preventive actions. As a result, economic loss can be reduced, and system safety can be ensured [25].

In this study, the gearbox dataset from Southeast University is adopted for experimental validation [26]. The data are collected from a drivetrain dynamics simulator (DDS) under the conditions of a rotational speed of 20 Hz and a load of 0 V. The dataset contains eight channels, including motor vibration, vibration of the planetary gearbox in the x-, y-, and z-directions, motor torque, and vibration of the parallel gearbox in the x-, y-, and z-directions [27]. The experiments cover five fault conditions: surface wear (Surface), root crack (Root), missing tooth (Miss), healthy condition (Healthy), and chipped tooth surface (Chipped) [28].

**Figure 3:**
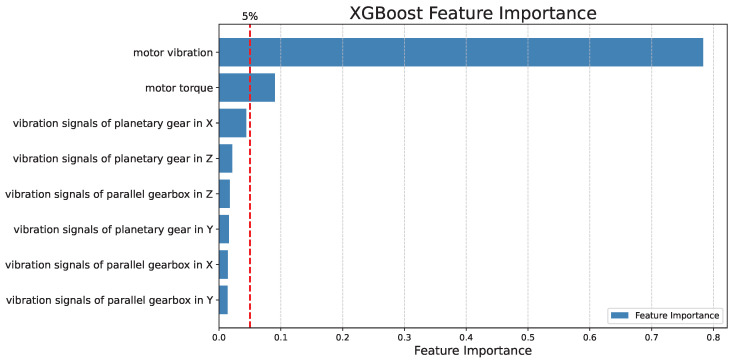
Order of importance of gearbox fault features.

To determine the input features of the model, XGBoost is used to rank the importance of the eight features, as shown in Figure 3. The results show that motor vibration and motor torque make the largest contributions to fault diagnosis, whereas the importance of the other channels is lower than 5%. Therefore, these two features are selected as the inputs of the SS-BRB model. In this study, representative low-dimensional features are intentionally adopted. The reason is that, in a standard BRB, the number of rules grows rapidly with the number of antecedent attributes and referential values. Directly introducing high-dimensional features may lead to severe rule explosion and reduced computational practicality. Therefore, feature selection is first performed to retain the most informative variables while preserving the interpretability and tractability of the BRB model.

The motor vibration ($X_{1}$) is described using five linguistic grades—very small (VS), medium (VM), rather large (VR), tremendous (VX), and extreme (VE). Likewise, the motor torque ($X_{2}$) is represented using three linguistic levels—low (L), medium (M), and high (H). The reference values of the attributes are listed in Table 1 and Table 2. The referential values of the results are given in Table 3. The initial belief distributions are presented in Table 4.

The gearbox dataset used in this study contains 5000 samples in total, with 1000 samples for each fault category, namely surface wear, root crack, missing tooth, healthy state, and chipped tooth. Among them, 1000 samples (200 samples per category) are used as the test set, while the remaining 4000 samples are used as the training set. Within the training set, 20% of the samples (800 samples) are treated as labeled samples and are used to initialize the BRB rule base, whereas the remaining 80% (3200 samples) are treated as unlabeled samples. For these unlabeled samples, high-reliability pseudo-labels are generated through the proposed dual-source evidence-driven mechanism and are then incorporated into the subsequent semi-supervised training process.

It should also be noted that the data are collected under a fixed operating condition (20 Hz, 0 V), which helps reduce the influence of operating-condition variation on the diagnostic features considered in this study. In addition, the original dataset does not provide ground-truth annotations of sensor noise levels. Therefore, this study does not assume any explicitly measured physical noise level for the gearbox data; instead, the robustness of the proposed method to noise is evaluated separately through controlled perturbation experiments. Moreover, since the samples are extracted from short observation windows under the same operating condition, the features used in this study can be regarded as relatively stable within each sample window for the purpose of model construction and evaluation. However, a formal statistical test of stationarity is beyond the scope of the present study.

### 5.2. Fault Diagnosis Analysis of SS-BRB

This section presents a comprehensive analysis of the fault diagnosis results of the proposed SS-BRB model. Section 5.2.1 focuses on the diagnostic performance of the model, Section 5.2.2 examines its generalization capability, and Section 5.2.3 further compares the proposed method with baseline methods and representative deep semi-supervised approaches.

The updated attribute reference values obtained after SS-BRB training are listed in Table 5 and Table 6. With the refinement and expansion of the antecedent reference values during training, the rule base is correspondingly expanded from 15 rules to 36 rules. The optimized rule weights and belief distributions of the updated rule base are presented in Table 7.

#### 5.2.1. Model Performance

**Figure 4:**
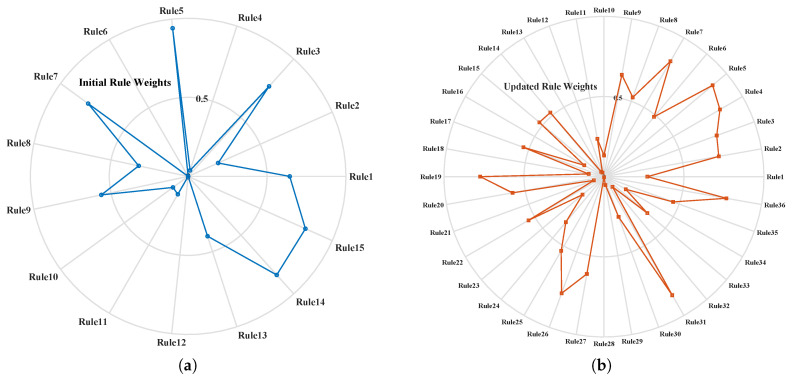
Comparison of rule weight variations before and after updating: (**a**) Initial rule weights; (**b**) Updated rule weights.

Figure 4a,b clearly show the optimization effect of the SS-BRB method on the BRB model structure in the gearbox fault diagnosis task. Because the initial model relies only on a limited number of labeled samples, the number of rules is restricted. In addition, some rule weights are equal to zero. As a result, the coverage of the model is insufficient, and the generalization ability is weak.

After high-quality pseudo-labels are introduced, the number of model rules is expanded to 36, and the weight distribution becomes more reasonable. The newly added rules can effectively capture the key fault patterns. At the same time, the weights of some original rules with high weights are adjusted dynamically. This result reflects the adaptive optimization capability of the model under the fusion of data-driven learning and knowledge-based reasoning.

**Figure 5:**
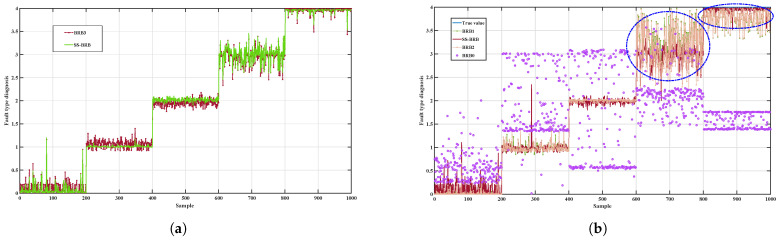
Comparison of SS-BRB with other BRBs on the gearbox dataset: (**a**) Comparison results between SS-BRB and BRB3; (**b**) Comparison of diagnostic performance between SS-BRB and other BRB variants.

Figure 5 shows the diagnostic accuracy of BRB1 (the BRB trained with a small number of labeled samples), BRB2 (the BRB trained with pseudo-labels obtained by k-nearest neighbors), SS-BRB, BRB3 (the BRB trained in a fully supervised manner), and BRB0 (the unoptimized BRB trained by semi-supervised learning) in the gearbox fault diagnosis task.

As can be seen from Figure 5a,b, the performance of the SS-BRB model is slightly lower than that of BRB3 trained in a fully supervised manner, but it is clearly better than that of BRB0, BRB1, and BRB2. In the region marked by the blue dashed circle, the prediction curve of SS-BRB almost overlaps with the true labels. Only small fluctuations are observed, and high stability is maintained. These results indicate good fitting ability and robustness. Especially in the stage with complex fault patterns, SS-BRB provides a clearer description of the boundaries between fault categories. In addition, the deviation caused by pseudo-label errors can be effectively suppressed. This result verifies its high reliability and application potential under semi-supervised conditions.

Table 8 presents a quantitative comparison of the performance metrics of different methods. SS-BRB achieves higher accuracy and lower MSE than the weakly supervised comparison methods BRB0–BRB2 while exhibiting performance close to that of the fully supervised BRB3. These results further verify the effectiveness of the dual-source evidence mechanism in improving pseudo-label quality and enhancing the stability of model training.

#### 5.2.2. Generalization Verification Experiments

In this experiment, the WD615 diesel engine dataset is collected under a steady-state operating condition, with a rotational speed of 1800 r/min and a sampling frequency of 12.8 kHz. The experimental settings include three operating conditions, namely the normal condition, the mild fault condition, and the severe fault condition [29]. Therefore, the present experiment evaluates the applicability of the proposed method to a different type of equipment under a fixed operating condition, rather than under varying operating regimes.

**Figure 6:**
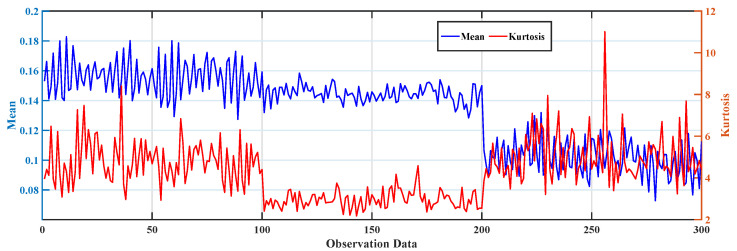
Trends of mean and kurtosis of WD615 diesel engine vibration signals.

To characterize the signal behavior during the evolution from normal operation to fault conditions, the mean and kurtosis are selected as input observation indicators in this study based on related research [30]. From a physical perspective, the mean reflects the overall amplitude level of the vibration signal, whereas kurtosis is more sensitive to impulsive components and abnormal fluctuations that are often associated with fault development. By combining these two indicators, both the overall variation trend and the abnormal impulsive behavior of the diesel engine vibration signal can be represented in a compact and interpretable manner. The corresponding data distribution is shown in Figure 6.

It should also be noted that the original dataset does not provide ground-truth annotations of sensor noise levels. Therefore, this study does not assume any explicitly measured physical noise level for the WD615 diesel engine data, and the robustness to noise is instead evaluated separately through controlled perturbation experiments in the subsequent analysis. Moreover, since the samples are extracted from short observation windows under the same steady-state operating condition, the statistical features used in this study can be regarded as relatively stable within each sample window for the purpose of model construction and evaluation. However, a formal statistical test of stationarity is beyond the scope of the present study.

**Figure 7:**
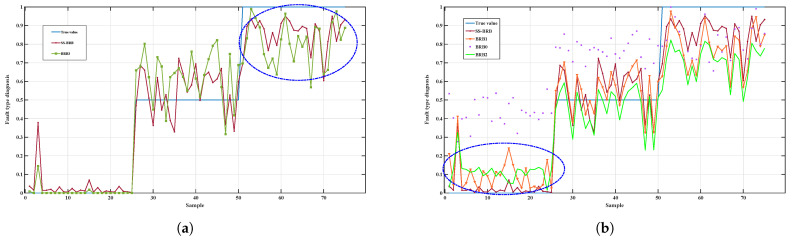
Comparison between SS-BRB and other BRBs on the WD615 dataset: (**a**) Comparison results between SS-BRB and BRB3; (**b**) Comparison between SS-BRB and other BRBs.

To further validate the applicability of the proposed SS-BRB model to different types of equipment, experiments are conducted on the WD615 diesel engine dataset in this study. The diagnostic results are compared with those of BRB0, BRB1, BRB2, and BRB3. The results of different methods are shown in Figure 7.

As shown in Figure 7, on the WD615 diesel engine dataset, the prediction results of SS-BRB are more consistent with the actual trend of state variation, which indicates better diagnostic accuracy and stability. In contrast, BRB1, BRB2, and BRB0 show different degrees of deviation in some sample intervals. More obvious fluctuations are observed, especially near the state transition regions. In particular, within the region marked by the blue dashed circle, SS-BRB tracks the state variation more accurately. This result indicates that the proposed method still has good adaptability to data from different types of equipment.

Table 9 shows that SS-BRB achieves superior overall performance on the WD615 diesel engine dataset. Its MSE is 0.0238, the lowest among all comparison methods, indicating the smallest deviation between predicted results and true states and the highest overall stability. Meanwhile, its accuracy reaches 92.3%, significantly higher than that of BRB0, BRB1, and BRB2 and only slightly lower than that of the fully supervised BRB3 at 94.6%. These results demonstrate that, under limited labeled samples, SS-BRB can effectively leverage information from unlabeled samples, achieving high diagnostic accuracy while maintaining better error control. This reflects its strong semi-supervised learning capability and generalization performance.

#### 5.2.3. Comprehensive Comparison with Baseline and Representative Deep Semi-Supervised Methods

To further evaluate the effectiveness of the proposed SS-BRB method, a comprehensive comparison was conducted with both traditional semi-supervised baselines, including label propagation (LP), label spreading (LS), and semi-supervised support vector machine (S^3^VM), and representative deep semi-supervised methods, namely Mean Teacher and FixMatch.

Table 10 summarizes the performance of different semi-supervised methods in terms of accuracy and Macro-F1. The traditional baselines, including LP and LS, exhibit poor performance, with accuracy values around 0.35 and Macro-F1 values below 0.35, indicating that these graph-based propagation methods are insufficient for handling the complex feature distributions and uncertainty in the considered fault diagnosis task. The S^3^VM achieves moderate improvement, with an accuracy of 0.5550 and a Macro-F1 of 0.5038, but its performance remains limited under scarce labeled data conditions.

The representative deep semi-supervised methods demonstrate clear performance gains over the traditional baselines. Mean Teacher achieves an accuracy of 0.7490 and a Macro-F1 of 0.7129, showing the benefit of consistency regularization in utilizing unlabeled samples. FixMatch further improves the results to 0.9700 in both accuracy and Macro-F1, confirming the effectiveness of confidence-based pseudo-labeling in semi-supervised fault diagnosis.

Nevertheless, the proposed SS-BRB achieves the best overall performance, with an accuracy of 0.9870 and a Macro-F1 of 0.9920. Compared with FixMatch, SS-BRB improves the accuracy by 1.7 percentage points and the Macro-F1 by 2.2 percentage points. These results indicate that the proposed method not only outperforms traditional semi-supervised baselines but also remains highly competitive against representative deep semi-supervised frameworks. More importantly, unlike conventional deep models, the proposed BRB-based framework preserves explicit rule-based reasoning and belief-distribution outputs, which provide better interpretability and transparency for safety-critical fault diagnosis under uncertain industrial conditions.

### 5.3. Effectiveness and Robustness Analysis of the DSE-PL Mechanism

In this section, Section 5.3.1 presents a visualization analysis of the DSE-PL mechanism. Section 5.3.2 discusses the effectiveness and robustness of the DSE-PL mechanism, Section 5.3.3 analyzes the impact of different labeled-to-unlabeled data ratios on model performance, and Section 5.3.4 further provides an ablation study on the dual-source evidence mechanism.

#### 5.3.1. Visualization Analysis of the DSE-PL Mechanism

To further validate the effectiveness of the proposed dual-source evidence fusion mechanism, the weight allocation process and the fusion results are visualized using the gearbox dataset.

**Figure 8:**
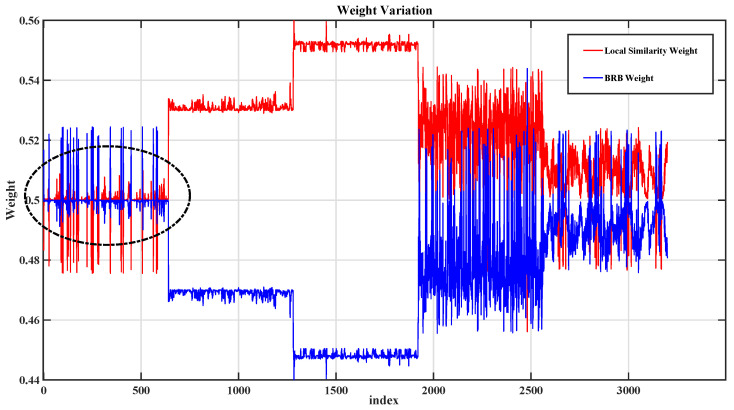
Variation trends of local similarity evidence and BRB-based global inference evidence.

Figure 8 illustrates the variation trends of the local similarity evidence and the BRB-based global inference evidence across different sample intervals. The black circle highlights the sample interval where the relative contributions of the two evidence sources change significantly. It can be observed that the two evidence sources exhibit complementary changes in their relative contributions to pseudo-label evaluation, which further reflects the dynamic nature of the proposed weighting mechanism. Specifically, the weights assigned to local evidence and global evidence are not fixed constants but are adaptively adjusted according to the relative reliability of the two evidence sources for each sample. As a result, in some sample intervals, local neighborhood information receives a larger weight and provides stronger support for pseudo-label assessment, whereas in other intervals, BRB-based global inference is assigned a higher weight and offers more informative guidance. This result indicates that different samples require different balances between data-driven local consistency and knowledge-driven global rule support. Therefore, the proposed dual-source mechanism can dynamically coordinate local and global evidence, thereby improving the reliability, flexibility, and rationality of pseudo-label assignment.

**Figure 9:**
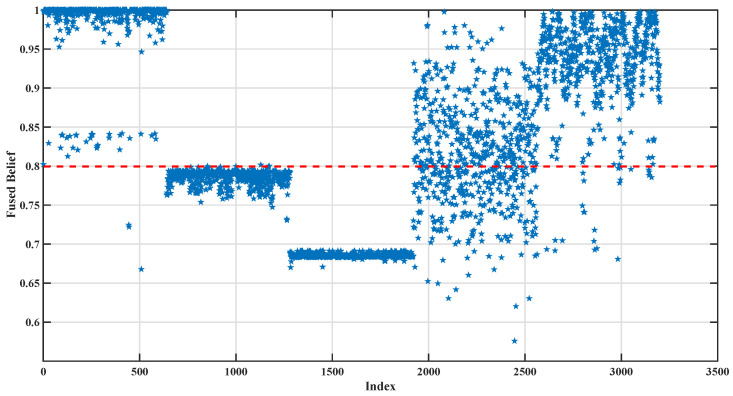
Distribution of confidence after fusion.

Figure 9 further illustrates the distribution of pseudo-label confidence after fusion. The red line denotes the preset confidence threshold of 0.80, and the stars indicate the samples with confidence values below this threshold. It can be seen that a large proportion of samples have final confidence values concentrated above or near the preset threshold of 0.80, while only a relatively small number of samples fall below this threshold. This indicates that the proposed mechanism can provide generally reliable pseudo-labels during the selection stage, thereby supporting the stable training of the SS-BRB model.

In summary, the visualization results verify the effectiveness of the proposed dual-source evidence mechanism from two aspects: complementary evidence coordination and high-confidence pseudo-label selection. The results show that local similarity evidence and BRB-based global inference evidence provide different but complementary support for pseudo-label evaluation under different sample characteristics. While preserving the interpretability of the BRB, the proposed mechanism improves pseudo-label reliability and enhances the diagnostic performance of the model.

#### 5.3.2. Robustness Assessment of DSE-PL on Different Datasets

To evaluate the effectiveness of different pseudo-label assignment methods, this study compares the Gaussian mixture model (GMM), density-based spatial clustering of applications with noise (DBSCAN), K-means clustering, k-nearest neighbors (KNN), and the proposed dual-source evidence-driven pseudo-label mechanism (DSE-PL). The comparison results on the gearbox and diesel engine datasets are presented in Table 8. The methods are quantitatively evaluated in terms of their ability to correctly assign unlabeled samples to the corresponding classes using the adjusted Rand index (ARI) and recall [31].

As shown in Table 11, the proposed DSE-PL mechanism achieves strong performance on both the gearbox and diesel engine datasets. On the gearbox dataset, DSE-PL attains an ARI of 0.9577 and a recall of 0.9828, demonstrating competitive performance that is slightly lower than that of KNN but substantially better than that of K-means, GMM, and DBSCAN. On the WD615 diesel engine dataset, DSE-PL achieves the best performance among all compared methods, with an ARI of 0.8176 and a recall of 0.9333, clearly outperforming KNN, K-means, GMM, and DBSCAN.

These results validate the effectiveness of DSE-PL. By integrating local similarity information with BRB-based global inference, the proposed method improves the reliability of pseudo-labels and enhances the stability and accuracy of semi-supervised training. Compared with distance-based or clustering-based pseudo-label generation methods, DSE-PL can assign unlabeled samples more accurately and reduce the adverse influence of noisy pseudo-labels.

In addition, KNN performs well on the gearbox dataset, but its ARI on the diesel engine dataset drops to 0.3652, indicating limited generalization ability under different equipment types and feature distributions. In contrast, DSE-PL maintains consistently high performance across both datasets, further demonstrating the advantage of the dual-source evidence mechanism in improving the generalization capability of the semi-supervised BRB model.

To further evaluate the robustness of the DSE-PL mechanism, this study compares the performance of different methods under various levels of noise. Random noise of 5%, 10%, and 15% is added, and Table 12 presents the ARI values under these different noise conditions.

As shown in Table 12, for both the gearbox and diesel engine datasets, the ARI of all methods decreases as the noise level increases (5%, 10%, 15%). However, the decline for DSE-PL is smaller than those for the other methods, indicating higher robustness. Notably, on the diesel engine dataset, DSE-PL achieves an ARI of 0.5793 even under 15% noise, significantly outperforming KNN, K-means, GMM, and DBSCAN. These results demonstrate that by integrating local similarity with BRB-based global inference, the dual-source evidence mechanism in DSE-PL effectively mitigates the noise interference in pseudo-label assignment, ensuring the stability and reliability of the semi-supervised BRB model in complex environments.

#### 5.3.3. Effect of Labeled Sample Proportion on Model Performance

To evaluate the impact of labeled-to-unlabeled sample ratios on SS-BRB performance, the proportion of labeled and unlabeled samples in the gearbox dataset is adjusted. The model performance under different data ratios is systematically analyzed. The comparison results for labeled data proportions of 10%, 20%, 30%, and 40% are shown in Table 13.

As the proportion of labeled samples increases from 10% to 30%, the model accuracy rises from 95% to 99.4%, and the MSE decreases significantly to 0.0227. This indicates that the introduction of high-reliability pseudo-labels substantially enhances the training signal, improving both the predictive accuracy and learning capability of the model.

Notably, when the labeled sample proportion increases from 30% to 40%, the model accuracy (98.5%) and MSE (0.0184) remain similar to the 30% condition. This suggests that the semi-supervised framework, combined with high-quality pseudo-labels generated by DSE-PL, can effectively leverage unlabeled data under limited labeled samples, achieving diagnostic performance comparable to that with a higher proportion of labeled data.

#### 5.3.4. Ablation Study on the Dual-Source Evidence Mechanism

To further verify the necessity of the dual-source evidence mechanism, a quantitative ablation study was conducted by comparing the full SS-BRB with two degraded variants, namely, using only local similarity evidence and using only BRB-based global inference evidence for pseudo-label evaluation.

As shown in Table 14, the local-similarity-only variant achieved an MSE of 0.0754, an accuracy of 91.5%, and a Macro-F1 of 0.7134, while the global-inference-only variant obtained an MSE of 0.0584, an accuracy of 92.4%, and a Macro-F1 of 0.7230. In contrast, the proposed dual-source SS-BRB achieved an MSE of 0.0189, an accuracy of 98.7%, and a Macro-F1 of 0.9920. Compared with the local-similarity-only variant, the proposed method reduces the MSE by 0.0565, improves the accuracy by 7.2 percentage points, and increases the Macro-F1 by 0.2786. Compared with the global-inference-only variant, it reduces the MSE by 0.0395, improves the accuracy by 6.3 percentage points, and increases the Macro-F1 by 0.2690. It can also be observed from Table 14 that the improvement in Macro-F1 is particularly significant, indicating that the dual-source mechanism not only improves overall diagnostic accuracy but also substantially enhances balanced diagnostic performance across different fault categories. These results clearly demonstrate that neither local similarity evidence nor global inference evidence alone is sufficient for robust pseudo-label evaluation, whereas their combination is essential for achieving both high accuracy and strong class-balanced diagnostic capability. Therefore, the necessity and distinct contribution of the proposed dual-source mechanism are well validated.

### 5.4. Summary of Experiments

The experiments in this section demonstrate that, to address the scarcity of labeled samples in complex industrial systems, the proposed SS-BRB method effectively integrates local similarity and BRB-based global inference through a dual-source evidence-driven pseudo-label mechanism, thereby improving the reliability of pseudo-labels for unlabeled samples. On the gearbox and WD615 diesel engine datasets, SS-BRB achieves high diagnostic accuracy and low MSE under limited labeled-data conditions, with performance close to that of fully supervised BRB models in the tested cases.

At the same time, dynamic weight adjustment and high-confidence selection effectively suppress the interference of noisy pseudo-labels, thereby enhancing training stability and model robustness. From a physical and modeling perspective, this behavior can be explained by the fact that the BRB framework represents fault knowledge through interpretable antecedent reference values and consequent belief distributions, while the dual-source mechanism further constrains pseudo-label selection using both local neighborhood consistency and global rule-based support. As a result, samples that are inconsistent with the local data structure or weakly supported by the current rule base are less likely to be incorporated as strong supervision, which helps the model capture relatively stable fault-related patterns rather than accidental noise. Moreover, SS-BRB shows good generalization under the tested datasets, fault categories, and labeled-to-unlabeled sample-ratio settings. Even with a low proportion of labeled samples, it can fully leverage unlabeled data to achieve diagnostic performance comparable to higher labeled proportions, validating its effectiveness under semi-supervised conditions and its applicability to fault diagnosis in complex industrial systems within the considered experimental settings.

## 6. Conclusions

In this paper, a dual-source evidence-driven semi-supervised belief rule base method is proposed to address the degradation of BRB fault diagnosis performance under limited labeled data conditions. By integrating local similarity evidence with BRB-based global inference, the proposed method improves the reliability of pseudo-labels for unlabeled samples and enables the effective utilization of unlabeled data while preserving the interpretability and inference transparency of the BRB framework. In addition, the incorporation of high-confidence pseudo-labeled samples into model training further enhances the rule learning capability and diagnostic performance of the BRB model.

Experimental studies on the gearbox dataset and the WD615 diesel engine dataset demonstrate the effectiveness of the proposed method. Under scarce labeled data conditions, the proposed SS-BRB achieves strong overall performance, with an Accuracy of 0.9870 and a Macro-F1 of 0.9920 in the main comparative experiment. In the ablation study, compared with the local-similarity-only variant, the proposed dual-source mechanism reduces the MSE by 0.0565, improves the Accuracy by 7.2 percentage points, and increases the Macro-F1 by 0.2786; compared with the global-inference-only variant, it reduces the MSE by 0.0395, improves the Accuracy by 6.3 percentage points, and increases the Macro-F1 by 0.2690. These results verify that the proposed dual-source evidence fusion mechanism can effectively improve pseudo-label quality and enhance the semi-supervised learning ability of the BRB model for fault diagnosis tasks.

From a broader perspective, the proposed framework is meaningful not only because it improves diagnostic performance under scarce labeled data but also because it provides an interpretable semi-supervised diagnosis framework for reliability engineering. In contrast to many black-box semi-supervised models, the proposed SS-BRB retains explicit rule-based reasoning and belief-distribution outputs, which are valuable for uncertainty-aware fault reasoning, engineering interpretation, and reliability-oriented maintenance decision support in complex industrial systems. Moreover, the experimental results indicate that the proposed method shows good robustness and good generalization under the tested datasets, fault categories, and labeled-to-unlabeled sample-ratio settings.

Nevertheless, several limitations remain in the current study. First, the effectiveness of the proposed method has so far been verified mainly on limited datasets and relatively typical operating conditions. Its adaptability to more complex operating environments, additional fault types, and higher-dimensional heterogeneous feature scenarios still requires further investigation. In particular, the current experiments mainly adopt representative low-dimensional features. Although this setting is suitable for validating the proposed semi-supervised BRB mechanism, directly introducing high-dimensional heterogeneous features into a standard BRB may lead to combinatorial rule explosion due to the rapid growth of antecedent combinations and referential values. Therefore, the scalability of the proposed framework in high-dimensional scenarios remains an important issue for further study.

Second, the initialization of the rule base and attribute reference values still depends to some extent on prior knowledge. It should also be noted that the proposed SS-BRB is a reconfigurable diagnosis framework rather than a fixed rule model for a specific device. When the diagnostic target changes, the core semi-supervised learning mechanism remains unchanged, while the task-specific BRB structure needs to be reconstructed according to the new equipment. In practice, this reconstruction includes selecting informative monitoring attributes, defining initial semantic reference values, initializing the rule base with a small number of labeled samples, and then further optimizing the model through the proposed dual-source evidence-driven semi-supervised learning process. Therefore, expert knowledge mainly serves as an interpretable initialization prior, whereas the final model is adaptively refined through labeled and unlabeled data.

Third, the current optimization strategy based on P-CMA-ES is mainly designed for offline parameter training rather than strict real-time continuous model updating. As a result, the present framework is more suitable for an offline-training and online-inference deployment mode. For practical industrial applications, a feasible extension is to decouple online diagnosis from model updating, that is, to perform real-time fault diagnosis using the currently trained SS-BRB model, while executing periodic or event-triggered background re-optimization based on newly accumulated labeled or high-confidence pseudo-labeled samples. However, such an online adaptation mechanism has not been fully implemented or validated in the current study and remains an important topic for future research.

Future work will therefore focus on three aspects: (1) incorporating multi-source heterogeneous signals such as vibration, acoustic emission, temperature, and current information to improve diagnosis under complex industrial conditions; (2) developing adaptive rule-base construction and update strategies, as well as scalable BRB variants combined with feature selection or dimensionality reduction techniques, to reduce expert dependence and improve transferability across different equipment; and (3) exploring lightweight incremental optimization and online diagnosis frameworks for real-time industrial applications. These efforts are expected to further improve the generality, scalability, and practical applicability of the proposed SS-BRB method in reliability engineering and intelligent fault diagnosis.

## Figures and Tables

**Table 1:** The reference values of $X_{1}$.

Attribute	Weight	VS	VM	VR	VX	VE
$X_{1}$	1	−0.7	−0.4267	−0.144	−0.1239	−0.09

**Table 2:** The reference values of $X_{2}$.

Attribute	Weight	L	M	H
$X_{2}$	0.9	−0.06	0.0117	0.085

**Table 3:** Reference values for fault diagnosis results.

Reference Points	Surface	Root	Miss	Healthy	Chipped
Reference value	0	1	2	3	4

**Table 4:** Initial belief rules.

Rule Number	Weight	$X_{1}$	$X_{2}$	The Belief Distribution
1	0.6437	VS	L	{0.0056, 0.3989, 0.1894, 0.1597, 0.2464}
2	0.2079	VS	M	{0.3110, 0.0371, 0.0266, 0.5519, 0.0733}
3	0.7663	VS	H	{0.2512, 0.0036, 0.0735, 0.0080, 0.6638}
4	0.0399	VM	L	{0.1950, 0.2919, 0.1142, 0.1598, 0.2391}
5	0.9422	VM	M	{0.0000, 0.0015, 0.0012, 0.0000, 0.9990}
6	0.0000	VM	H	{0.2864, 0.2048, 0.0599, 0.2980, 0.1512}
7	0.7835	VR	L	{0.2151, 0.4640, 0.2234, 0.0859, 0.0115}
8	0.3203	VR	M	{0.9570, 0.0117, 0.0068, 0.0183, 0.0062}
9	0.5625	VR	H	{0.2722, 0.1263, 0.6028, 0.0000, 0.0033}
10	0.1188	VX	L	{0.2175, 0.0051, 0.2396, 0.4000, 0.1378}
11	0.1307	VX	M	{0.0261, 0.1634, 0.0010, 0.3385, 0.4620}
12	0.0000	VX	H	{0.3762, 0.0019, 0.0702, 0.5325, 0.0192}
13	0.3976	VE	L	{0.3170, 0.0196, 0.1414, 0.2310, 0.2910}
14	0.8389	VE	M	{0.0657, 0.2230, 0.4811, 0.1101, 0.1201}
15	0.8136	VE	H	{0.4667, 0.0117, 0.1310, 0.1085, 0.2820}

**Table 5:** Updated reference values of $X_{1}$.

Attribute	Weight	VS	VM	VN	VR	VX	VE
$X_{1}$	0.9923	−0.7	−0.4267	−0.1664	−0.1444	−0.1239	−0.09

**Table 6:** Updated reference values of $X_{2}$.

Attribute	Weight	Z	X	L	N	M	H
$X_{2}$	0.2878	−0.06	−0.0030	0.0043	0.0447	0.0653	0.085

**Table 7:** Updated belief distributions.

Rule Number	Weight	$X_{1}$	$X_{2}$	The Belief Distribution
1	0.2716	VS	Z	{0.1970, 0.1123, 0.2544, 0.3060, 0.1302}
2	0.7286	VS	X	{0.1754, 0.5649, 0.0996, 0.0169, 0.1431}
3	0.7500	VS	L	{0.2761, 0.1069, 0.0939, 0.2369, 0.2861}
4	0.8371	VS	N	{0.0344, 0.0839, 0.4863, 0.0660, 0.3293}
5	0.8866	VS	M	{0.2697, 0.0730, 0.2650, 0.1893, 0.2020}
6	0.4873	VS	H	{0.1604, 0.3376, 0.0603, 0.0432, 0.3385}
7	0.8311	VM	Z	{0.6153, 0.1345, 0.0166, 0.1243, 0.1093}
8	0.5265	VM	X	{0.3238, 0.2415, 0.2784, 0.1297, 0.0266}
9	0.6446	VM	L	{0.7088, 0.0801, 0.0597, 0.1008, 0.0507}
10	0.1328	VM	N	{0.4562, 0.0375, 0.2139, 0.1182, 0.1741}
11	0.2400	VM	M	{0.2174, 0.0932, 0.0303, 0.1814, 0.4778}
12	0.0271	VM	H	{0.4516, 0.2448, 0.1158, 0.0584, 0.1294}
13	0.0362	VN	Z	{0.0054, 0.0070, 0.0051, 0.0100, 0.9715}
14	0.5220	VN	X	{0.1331, 0.1113, 0.2072, 0.0482, 0.5002}
15	0.5272	VN	L	{0.0007, 0.0007, 0.0047, 0.0007, 0.9932}
16	0.1420	VN	N	{0.4687, 0.1269, 0.1076, 0.1177, 0.1792}
17	0.5359	VN	M	{0.4349, 0.1163, 0.1894, 0.0287, 0.2307}
18	0.0972	VN	H	{0.0371, 0.2132, 0.1234, 0.5108, 0.1155}
19	0.7732	VR	Z	{0.0614, 0.2326, 0.0365, 0.3628, 0.3038}
20	0.5806	VR	X	{0.0026, 0.1727, 0.2247, 0.0359, 0.5641}
21	0.0668	VR	L	{0.2758, 0.0236, 0.0581, 0.2617, 0.3807}
22	0.5446	VR	N	{0.1155, 0.0775, 0.1206, 0.0424, 0.6439}
23	0.1743	VR	M	{0.1293, 0.0454, 0.0675, 0.6399, 0.1178}
24	0.3707	VR	H	{0.2990, 0.0868, 0.4247, 0.0550, 0.1344}
25	0.5347	VX	Z	{0.9301, 0.0192, 0.0066, 0.0129, 0.0312}
26	0.7742	VX	X	{0.1288, 0.3306, 0.5176, 0.0004, 0.0226}
27	0.6160	VX	L	{0.9973, 0.0002, 0.0065, 0.0000, 0.0000}
28	0.0033	VX	N	{0.5403, 0.0273, 0.1470, 0.0908, 0.1945}
29	0.0531	VX	M	{0.3575, 0.1138, 0.1123, 0.1300, 0.2863}
30	0.2672	VX	H	{0.1901, 0.0672, 0.3249, 0.3587, 0.0590}
31	0.8535	VE	Z	{0.3857, 0.0812, 0.4385, 0.0439, 0.0507}
32	0.0822	VE	X	{0.1382, 0.0204, 0.1921, 0.1228, 0.5266}
33	0.3533	VE	L	{0.0465, 0.3603, 0.0515, 0.0866, 0.4551}
34	0.1571	VE	N	{0.3287, 0.2925, 0.1224, 0.2478, 0.0088}
35	0.4601	VE	M	{0.2381, 0.2977, 0.0354, 0.0475, 0.3813}
36	0.7768	VE	H	{0.4069, 0.1239, 0.0847, 0.2017, 0.1829}

**Table 8:** Comparative performance of SS-BRB and other BRBs on the gearbox dataset.

Metric	MSE	Accuracy
BRB0	1.9669	22.2%
BRB1	0.0624	91.8%
BRB2	0.0638	91%
BRB3	0.0119	99.7%
SS-BRB	0.0189	98.7%

**Table 9:** Performance comparison of SS-BRB and other BRBs on the WD615 dataset.

Metric	MSE	Accuracy
BRB0	0.1089	87.2%
BRB1	0.0352	85%
BRB2	0.0351	84%
BRB3	0.0282	94.6%
SS-BRB	0.0238	92.3%

**Table 10:** Accuracy and Macro-F1 results of different semi-supervised methods.

Method	Accuracy	Macro-F1
LP	0.3500	0.3478
LS	0.3430	0.3405
S^3^VM	0.5550	0.5038
Mean Teacher	0.7490	0.7129
FixMatch	0.9700	0.9700
SS-BRB	0.9870	0.9920

**Table 11:** Recall and ARI for each method on different datasets.

Method	Gearbox		WD615 Diesel Engine	
	ARI	Recall	ARI	Recall
DSE-PL	0.9577	0.9828	0.8176	0.9333
KNN	0.9891	0.9956	0.3652	0.6933
K-means	0.7686	0.2044	0.3238	0.1267
GMM	0.7298	0.5541	0.4490	0.3733
DBSCAN	0.0000	0.2000	0.0004	0.3200

**Table 12:** ARI of DSE-PL with other methods under different disturbance intensities.

Perturbation Intensities	Gearbox			WD615 Diesel Engine		
	5%	10%	15%	5%	10%	15%
DSE-PL	0.8593	0.7837	0.6886	0.7425	0.6413	0.5793
KNN	0.8890	0.8078	0.7109	0.3377	0.3014	0.2437
K-means	0.4491	0.2758	0.1197	0.3148	0.2984	0.2305
GMM	0.3720	0.2243	0.1186	0.3709	0.2987	0.1721
DBSCAN	0.0000	0.0000	0.0000	0.0020	0.0009	0.0001

**Table 13:** MSE and accuracy for different ratios of labeled to unlabeled data.

Proportion of Labeled Samples	MSE	Accuracy
10%	0.0554	95%
20%	0.0189	98.7%
30%	0.0227	99.4%
40%	0.0184	98.5%

**Table 14:** Quantitative ablation results of different evidence sources in pseudo-label evaluation.

Method	MSE	Accuracy	Macro-F1
Local similarity only	0.0754	91.5%	0.7134
Global inference only	0.0584	92.4%	0.7230
SS-BRB	0.0189	98.7%	0.9920

## Data Availability

The WD615 diesel dataset that has been used is confidential. The SEU gearbox dataset that supports the findings of this study is available at https://github.com/Yxz3930/SEU-datasets (accessed on 10 February 2026).
